# Translation and Validation of the Arabic Version of the Cerebral Palsy-Quality of Life Child Self-Reported Questionnaire Among Children in the Saudi Arabian Population

**DOI:** 10.7759/cureus.69500

**Published:** 2024-09-16

**Authors:** Wadha Alsebeai, Amira M Youssef, Waad Alzubaidi, Deem Alfedaih

**Affiliations:** 1 Research and Scientific Center, Sultan Bin Abdulaziz Humanitarian City, Riyadh, SAU; 2 Medicine, Nora Bint Abdelrahman University, Riyadh, SAU

**Keywords:** cerebral palsy, cerebral palsy in children, cerebral palsy-quality of life child self-reported questionnaire, cp-qol child self-report questionnaire, cross-sectional study, quality of life, questionnaire translation and adaptation, rehablitation, translation and cultural adaptation

## Abstract

Objectives: This study aimed to assess the psychometric properties of the Cerebral Palsy-Quality of Life Child Self-Reported (CP-QoL Child Self-Report) questionnaire after it was translated and culturally adapted into Arabic.

Methods: This is a cross-sectional study that was conducted during the period from February 2021 to June 2023 in Sultan Bin Abdulaziz Humanitarian City (SBAHC) and the Children with Disability Association in Riyadh, Saudi Arabia, on children with cerebral palsy (CP) between the ages of nine to 12 years. After translation and cross-cultural adaptation, the questionnaire was administered to 65 children. The reliability and internal consistency of the tool were assessed using the intraclass correlation coefficient (ICC) and Cronbach’s alpha. The validity was analyzed through the association between the CP-QoL Child Self-Report questionnaire and the Arabic version of the Pediatric Quality of Life Inventory (PedsQL) 3.0-CP module (Mapi Research Trust, Lyon, France) using Pearson’s correlation coefficient.

Results: The overall internal consistency was 0.870, where it ranged from 0.616 for the pain and impact of disability domain to 0.748 for the social well-being and acceptance domain. The interobserver (test-retest) reliability ranged from 0.540 to 0.779. There was a weak correlation between the domains of the CP-QoL Child Self-Report questionnaire and different domains of PedsQL-CP module. The overall quality of life (QoL) score of the children with CP who participated in the current study was 57.86±4.97.

Conclusions: The result of the current study suggests that the English version of the CP-QoL Child Self-Report questionnaire was effectively translated into Arabic and had good psychometric properties in evaluating the QoL of Saudi children with CP aged between nine and 12 years.

## Introduction

The term cerebral palsy (CP) describes a collection of diseases with varying degrees of severity that share key developmental characteristics rather than a single disease entity [1]. Cerebral palsy describes a group of permanent disorders of the development of movement and posture, causing activity limitation, that is attributed to nonprogressive disturbances that occur in the developing fetal or infant brain [2]. As per the American Cerebral Palsy Academy, CP has four motor types, including spastic, dyskinetic, ataxic, and hypotonic [3].

Cerebral palsy is the most common motor disability in childhood. Population-based studies from around the world report that the prevalence estimates of CP range from 1.5 to more than four per 1,000 live births or children [4-8]. In the Kingdom of Saudi Arabia, CP is the most common neurologic disorder among Saudi children, with a prevalence rate of 23.4/10 000 [9].

Cerebral palsy is a lifelong condition that affects not only the individual and the family but also the community [10]. It imposes a high social and financial burden on the patients, their families, and the health system. This burden is due to both mortality and a higher rate of disabilities and complications. Such a burden consequently affects the quality of life (QoL) of the patients and their families, as reported by a systematic review, where the QoL was significantly impaired among patients with CP compared to their normal peers [11]. Therefore, many efforts were directed to develop valid and reliable questionnaires to assess QoL among this group of children.

Even though there are several questionnaires available to assess the QoL in children [12], there are a limited number of condition-specific tools to measure the QoL among children with CP. This includes the DISABKIDS CP Module, the Caregiver Priorities and Child Health Index of Life with Disabilities (CPCHILD), and the Pediatric Quality of Life Inventory (PedsQL)-CP module (Mapi Research Trust, Lyon, France) [13,14]. However, those three questionnaires are limited by lacking the ability to measure the feeling of life among CP patients. The Cerebral Palsy-Quality of Life Child Self-Reported (CP-QoL child self-report) questionnaire is the first QoL questionnaire designed for children with CP to assess their well-being. It has two primary forms, both for caregivers and children [15]. It has been found reliable and valid in different languages besides English [16-18].

Even though CP is the most common neurologic disorder among Saudi children in the Kingdom of Saudi Arabia, to the best of our knowledge, data concerning the QoL among patients with CP are lacking. From this perspective, and as per the recommendations of the WHO regarding the translation and cultural adaptation of existing tools, the current study aims to translate the CP-QoL Child Self-Report questionnaire into the Arabic language and determine its psychometric properties among children aged between nine and 12 years in Saudi Arabia.

## Materials and methods

Study design and participants

This is a cross-sectional study that was conducted during the period from February 2021 to June 2023 in Sultan Bin Abdulaziz Humanitarian City (SBAHC) and the Children with Disability Association in Riyadh, Saudi Arabia, using a paper-based self-administered CP-QoL Child Self-Report questionnaire that had 52 questions. The study included children aged between nine and 12 years with a confirmed CP diagnosis. Subjects who had received a botulinum toxin A injection or who had undergone any surgical procedures six months before the study were excluded.

The CP-QoL Child Self-Report questionnaire

The CP-QoL Child Self-Report questionnaire assesses several aspects of a child’s life, including physical well-being, social well-being, emotional well-being, school, access to services, and acceptance by others. Almost all the items have a nine-point rating scale, where 1 = very unhappy, 3 = unhappy, 5 = neither happy nor unhappy, 7 = happy, and 9 = very happy.

Study phases

The study was conducted in three phases: translation and cross-cultural adaptation, pre-testing, and data collection.

Phase One: Translation and Cross-Cultural Adaptation

The process of translation and cross-cultural adaptation followed the international recommendations, including translation into Arabic, reconciled translation, back translation, final translation, pre-test, and cultural adaptation. Firstly, two translators independently translated the CP-QoL Child Self-Report questionnaire (nine to 12 years) from English into Arabic. Both translators were fluent in English and Arabic, with Arabic as their native language. Secondly, both translations were compared, resulting in a reconciled translation that consisted of a consensual version with item adequacy and reconciliation. At this stage, to assess face validity, a team of researchers who had experience with children with CP and adolescents were requested to analyze item by item, choose the best translation, and suggest another translation if necessary. Then, an English translator who is fluent in Arabic was asked to carry out the back-translation, that is, translate the reconciled translation into the English language, which was sent to the authors of the original questionnaire to identify and correct the discrepancies regarding the conceptual equivalence. The fourth step included the review and comparison of the back-translated version corrected by the questionnaire authors with the original version in English that generated the final translation, which was used in the pre-test.

Phase Two: Pre-test

In the pre-test phase, the final version of the questionnaire was applied to 10 patients with CP to verify whether all items were comprehensible and satisfactory. To test the cultural equivalence, the questions that did not lead to a good understanding were discussed once again, reformulated by the researchers, and applied to another group of 10, until all items of the questionnaire were understood by 90% of the respondents. The average time needed to complete the questionnaire ranged from 15 to 25 minutes.

Phase Three: Data Collection 

After the pre-test, the data collection was conducted through a systematic random sampling technique. A list of all eligible patients who were visiting the outpatient clinics or admitted to the inpatient department was prepared, and patients were selected randomly to participate in the study.

Instruments

Besides the Arabic versions of the CP-QoL Child Self-Report questionnaire as per the validation protocol, the Arabic version of the PedsQL 3.0-CP module (Mapi Research Trust, Lyon, France) was also considered. The PedsQL 3.0-CP is a 35-item module that encompasses seven scales: (1) daily activities (nine items); (2) school activities (four items); (3) movement and balance (five items); (4) pain and hurt (four items); (5) fatigue (four items); (6) eating activities (five items); and (7) speech and communication (four items). It took about five minutes to administer the scale [19]. Additionally, the children’s sociodemographic and clinical information, including gender, age, and clinical history, was also collected.

Reliability

The reliability tests were assessed in terms of internal consistency sensitivity, and reproducibility.

Internal Consistency

Cronbach's alpha was used to evaluate the internal consistency of the translated questionnaire, and reliability indices were deemed satisfactory when they had values between 0.70 and 0.95.

Sensitivity

The sensitivity of the Arabic CP-QoL Child Self-Report questionnaire was assessed by evaluating the floor and ceiling effects where the percentage of minimal and maximal scores reached in each subscale. As per Terwee et al.'s study, ceiling or floor effects were considered if more than 15% of participants achieved the lowest or highest possible score [20].

Test-Retest Reliability

The test-retest method with a gap of 14 days, which entails answering the questionnaire twice, was used to assess reproducibility by evaluating the intra-class correlation coeﬃcient (ICC). The gap between the tests (14 days) was sufficient to rule out the memory effect but not long enough to prevent improvements in quality of life. The ICC was considered excellent when ICC ≥ 0.75; satisfactory when 0.4 ≤ ICC < 0.75; and weak when ICC < 0.419.

Statistical analysis

Descriptive statistics were conducted, where categorical variables were presented as counts and proportions, while continuous variables were presented as mean and standard deviation (SD). The Chi-square test (χ2) was used for categorical data, while the unpaired t-test was used for numerical data. Pearson’s correlation coefficients were used to examine the correlation between the CP-QoL Child Self-Report questionnaire and the PedsQL-CP module. The strength of correlation was interpreted based on the following criteria: 0.00 to 0.25 as little or no relationship, 0.25 to 0.50 as a fair relationship, 0.50 to 0.75 as a moderate to good relationship, and above 0.75 as a good to excellent relationship [21]. The level of confidence was chosen to be at 95% (p < 0.05). IBM SPSS® Statistics software for Windows, version 26 (IBM Corp., Armonk, NY) was used for the statistical analysis. All QOL scores were converted to a scale from 0 to 100, i.e., 1 = 0, 2 = 12.5, and 3 = 25. The algebraic means of item values are computed for each domain.

Ethical considerations

The study was approved by the institutional review board at Sultan bin Abdulaziz Humanitarian City (approval number: 41-2021-IRB). It was compiled following the Declaration of Helsinki, the International Conference on Harmonization of Good Clinical Practice, and the local regulations for clinical research.

## Results

As shown in Table 1, the mean age of the study cohort was 10.6±1.08 years. Males constituted 37 participants (56.92%) among the total sample, compared to 28 (43.08%) for females. The majority of the children were attending normal schools at 63 (96.93%), and Gross Motor Function Classification System (GMFCS) levels II and III predominated other types at 21 (32.31%) and 26 (40.00%), respectively.

**Table 1:** Demographic characteristics of the studied group (N = 65) GMFCS: Gross Motor Function Classification Scale

Characteristics	Frequency
Age (Mean ± SD), years	10.6 ± 1.08
Gender n (%)	
Boys	37 (56.92)
Girls	28 (43.08)
Education n (%)	
Normal school	63 (96.93)
Special school	2 (3.07)
Place of residence n (%)	
Urban	60 (92.31)
Rural	5 (7.69)
GMFCS level n (%)	
I	6 (9.23)
II	21 (32.31)
III	26 (40)
IV	6 (9.23)
V	6 (9.23)

Reliability

Internal Consistency

The reliability of the CP-QoL Child Self-Report questionnaire (nine to 12 years) was acceptable with a Cronbach’s alpha overall score of 0.870, where it ranged from 0.616 for the pain and impact of disability domain to 0.748 for the social well-being and acceptance domain as shown in Table 2.

**Table 2:** Descriptive statistics and reliability of each domain and the entire CP-QoL child self-report questionnaire Values are expressed as mean ± SD; *Values between 0.70 and 0.90 indicate high reliability. CP-QoL Child Self-Report: Cerebral Palsy-Quality of Life Child Self-Reported questionnaire

CP-QoL Child Self-Report subscale	Mean score	Ceiling %	Flooring %	Cronbach’s alpha*
Overall score	57.86 ± 4.97	3.07 %	1.53%	0.870
Social well-being and acceptance	69.13 ± 8.27	13.84 %	1.53%	0.748
Feeling about functioning	67.90 ± 8.03	9.23%	1.53%	0.685
Participation and physical health	70.44 ± 7.76	20.0%	1.53%	0.699
Emotional well-being and self-esteem	64.03 ± 4.77	46.15 %	3.07%	0.730
Pain and impact of disability	17.77 ± 14.79	4.65%	18.46%	0.616

Sensitivity

The sensitivity of the Arabic CP-QoL Child Self-Report questionnaire was evaluated by assessing the floor and ceiling effects, where the percentage of minimal and maximal scores was reached in each subscale. Participation in physical health, emotional well-being, and self-esteem domains showed a ceiling effect, with 46.15% and 20.0% of children having the maximum score as shown in Table 2.

Test-Retest Reliability

The ICC was considered excellent for feeling about the function domain (ICC = 0.754) and emotional well-being and self-esteem (ICC = 0.779). The ICC was satisfactory for the social well-being and acceptance domain (ICC = 0.566), participation and physical health domain (ICC = 0.726), and pain and impact of disability domain (ICC = 0.540), as shown in Table 3.

**Table 3:** Test-retest reliability for the CP-QoL Child Self-Report questionnaire CP-QoL Child Self-Report: Cerebral Palsy-Quality of Life Child Self-Reported

CP-QoL Child Self-Report subscale	Intraclass correlation coefficient (ICC)
Overall score	0.719
Social well-being and acceptance	0.566
Feeling about functioning	0.754
Participation and physical health	0.726
Emotional well-being and self-esteem	0.779
Pain and impact of disability	0.540

Construct validity

The construct validity was analyzed using Pearson’s correlation coefficient to assess the relation between the relationship between the CP-QoL Child Self-Report questionnaire and the PedsQL-CP module. Table 4 shows a significant positive correlation between the CP-QoL Child Self-Report questionnaire's social and well-being and acceptance domains and the daily activities domain, the movement and balance domain, the pain and hurt domain, the fatigue domain, and the speech and communication domain. The feeling about the function domain was significantly correlated with the movement and balance domain and the fatigue domain. The participation and physical health domains were significantly correlated with the pain and hurt domain, fatigue domain, and speech and communication domain. The pain and impact of the disability domain were significantly negatively correlated with daily activities and eating activities. The majority of the correlations were of low and moderate magnitude. 

**Table 4:** Correlation between the domains of the CP-QoL Child Self-Reported questionnaire and the PedsQL-CP module **. Correlation is significant at the 0.01 level (two-tailed).*. Correlation is significant at the 0.05 level (two-tailed) GMFCS: Gross Motor Function Classification Scale; PedsQL-CP: Pediatric Quality of Life Inventory-Cerebral Palsy

PedsQL-CP module							
CP-QoL Child Self-Report questionnaire	Daily activities	School activities	Movement and balance	Pain and hurt	Fatigue	Eating activities	Speech and communication
Social well-being and acceptance	0.357^*^	0.159	0.342^*^	0.307^*^	0.296^*^	0.208	0.303^*^
Feeling about functioning	0.138	0.181	0.296^*^	0.18	0.294^*^	0.199	0.209
Participation and physical health	0.203	0.052	0.275	0.329^*^	0.365^**^	0.066	0.287^*^
Emotional well-being and self-esteem	-0.194	-0.207	-0.254	0.073	0.024	-0.228	0.062
Pain and impact of disability	-0.284^*^	-0.101	-0.138	0.052	-0.108	-0.367^**^	-0.153
Overall score	0.01	0.023	0.159	0.310^*^	0.247	-0.12	0.176

Quality of life

The overall QoL score of the children with CP who participated in the current study was 57.86±4.97, with the highest mean score for participation and physical health at 70.44±7.76 and the lowest for the pain and the impact of disability domain at 17.77±14.79 (Table 5). When the QoL was assessed according to sociodemographic characteristics, the mean QoL score was higher among males compared to females at 74.13±7.44 versus 69.33±8.66, respectively, with the difference being marginally significant with a p-value of 0.06. The QoL did not vary significantly across the type of school and place of residence of GMFCS levels (P = 0.913) as shown in Table 5.

**Table 5:** The mean overall score of the Cerebral Palsy-Quality of Life Child Self-Reported questionnaire and different characteristics of the study sample GMFCS: Gross Motor Function Classification Scale

Characteristics	Mean overall score (SD)	P-value
Gender		
Male	74.13 ± 7.44	0.06
Female	69.33 ± 8.66	
Education		
Normal school	72.10 ± 8.17	0.36
Special school	69.82 ± 15.71	
Place of residence		
Urban	71.81 ± 8.49	0.37
Rural	74.59 ± 5.21	
GMFCS level		
I	73.96 ±7.70	0.913
II	71.57 ± 7.87	
III	71.36 ± 9.90	
IV	76.32 ± 3.26	
V	71.02 ± 5.69	

The QoL within each domain was also assessed according to GMFCS levels, and there was no significant difference in all the domains of the QoL of the children according to different levels of GMFCS as shown in Table 6.

**Table 6:** Mean quality of life scores on the CP-QoL Child Self-Report questionnaire depending on the GMFCS level CP-QoL Child Self-Report: Cerebral Palsy-Quality of Life Child Self-Reported; GMFCS: Gross Motor Function Classification Scale

GMFCS level						
	I	II	III	IV	V	P-value
Social well-being and acceptance	69.62 ± 8.70	67.03 ± 10.75	69.78 ± 7.32	72.67 ± 2.89	70.35 ± 4.04	0.629
Feeling about functioning	69.79 ± 5.23	68.08 ± 8.02	67.07 ± 8.87	72.08 ± 3.49	65.59 ± 9.74	0.66
Participation and physical health	72.35 ± 7.28	71.59 ± 7.84	69.41 ± 7.80	71.36 ± 6.30	68.30 ± 10.19	0.78
Emotional well-being and self-esteem	64.73 ± 2.37	64.27 ± 5.88	63.60 ± 5.08	65.80 ± 1.88	62.94 ± 2.37	0.85
Pain and impact of disability	15.19 ± 21.02	19.70 ± 12.84	17.44 ± 15.37	17.60 ± 17.65	15.18 ± 13.99	0.95
Overall score	58.33 ± 4.22	58.13 ± 4.50	57.46 ± 6.09	59.90 ± 3.74	56.47 ± 3.05	0.82

## Discussion

The current study aimed to translate the English version of the CP-QoL Child Self-Report questionnaire into the Arabic language and to assess the psychometric properties of the translated version. The researchers followed the expert recommendations in their methodology [22] and made sure that the Arabic-translated version was equivalent to the English version and culturally adaptable to the Saudi community. The majority of the children reported that they were able to complete the questionnaire with no or minimal help, which indicates that the expressions used in the Arabic translation were clear and easily understood by the target population of children with CP. Therefore, no additional cultural adaptation was needed during this study.

Despite the availability of the Arabic version of the primary caregiver form of the CP QoL questionnaire for children aged between four and 12 years, many researchers highlighted the importance of validating the questionnaire for the children through a self-report form. This could be mainly due to different degrees of perceptions of the quality of life between the children and their adult caregivers. This is not to mention that the correlation between the child’s self-assessment and the perception of their caregivers was reported to be low [23].

In the current translation, the overall Cronbach’s alpha was 0.870, which indicates excellent internal consistency. The Cronbach’s alpha values across different domains of the translated Arabic version of the CP-QoL Child Self-Report questionnaire ranged from 0.616 to 0.748, which is within the range of the original English questionnaire values of 0.80-0.90 [24], and other languages versions such as the Chinese version with Cronbach’s alpha values of 0.84-0.89 [17]. In the current translation, only the pain and impact of disability domain had an acceptable internal consistency of 0.61. This was also observed in other versions of the questionnaire, such as the Turkish and Persian versions [18,25].

Test-retest reliability of the Arabic version of CP-QoL Child Self-Report questionnaire (ICC = 0.540-0.779) falls in the range of the original English questionnaire (ICC = 0.76-0.89, with 53 participants) and other countries' language versions, such as the Chinese version (ICC = 0.74 to 0.92, with 44 participants) [17], and the Brazilian version (ICC = 0.41-0.89, with 65 participants) [26]. The lowest value obtained for the ICC for test-retest reliability in the current study was for the pain and impact of disability domain (ICC = 0.540). This was observed also by other researchers with different language versions of the questionnaire and could be because this domain is related to a subjective state that could change over time between the first and second assessments.

Concerning construct validity, there was a weak correlation between the domains of the CP-QoL Child Self-Report questionnaire and the different domains of the PedsQL-CP module. The main reason could be the conceptual differences between the two measures of quality of life. The CP-QoL Child Self-Report questionnaire measures perspectives of quality of life specific to CP, whereas the generic PedsQL-CP module measures the frequencies of the problems and the difficulties that children experience. This was also observed by Prasertsukdee et al. while assessing the validity of the Thai version of the CP-QoL Child Self-Report questionnaire [27]. Additionally, the Turkish version reported a weak correlation between the CP-QoL Child Self-Report and the Health-Related Quality of Life questionnaire for Children (Kid-KINDL). Such a weak correlation between the CP-QoL Child Self-Report questionnaire and other QoL questionnaires could be supported by the explanation that those questionnaires being generic QoL questionnaires are less sensitive compared to CP-QoL Child Self-Report questionnaire as a disease-specific instrument [24].

Cerebral palsy is a lifetime disability that affects the quality of life of children and their families. As per the Global Burden of Disease Pediatrics, it is recommended to measure the quality of life of CP patients to enable healthcare professionals to intervene effectively for better outcomes [28]. Therefore, the current study also aimed to assess the QoL of children with CP using the CP-QoL Child Self-Report questionnaire (nine to 12 years), which is a condition-specific QoL instrument that assesses how the child feels about different aspects of his or her life. The overall mean QoL in our study was 57.86±4.97. This was higher than the mean score among Indian children at 7.36±5.30 [29]. This was also observed in all the QoL domains, except for the pain and impact of disability domain, which was lower among the Saudi children at 17.77±14.79 compared to 24.06±5.15 among the Indian children [29]. On the other hand, the mean scores of different domains of QoL among children with CP were lower than what was reported among Australian children [30]. In different language versions of the questionnaire, the pain and impact of disability domain had the lowest mean QoL score, which is an expected finding since pain is negatively correlated with the quality of life of CP patients [31].

In the present study, QoL was not affected by gender. This finding was in accordance with that of another study among Indian [29] and Malaysian children with CP [32]. Despite the influence of female hormones on the ability to reduce the consequences of brain damage and consequently improve the QoL [33], previous studies have reported that females usually have worse QoL compared to males [34].

Similarly, the mean overall score of QoL did not vary significantly across different GMFCS categories. This was also observed among different populations, such as Indian [28], Japanese [35], and Korean children [36]. This is in line with the finding of Iranian researchers that the gross motor function level cannot be used as a predictor of QoL for children with CP [37]. This sheds light on the importance of taking other factors that could affect the QoL of children with CP, such as cultural, environmental, economic, and social factors, into account while aiming to improve the QoL of such patients.

The study had several limitations, especially regarding the lack of additional data on the socioeconomic characteristics. Additionally, the sample size was relatively small; however, it was comparable to previous studies in the different versions such as the Brazilian-Portuguese version (N = 65), the English version (N = 53), and the Chinese version (N = 44). Another limitation of this study was the difficulty in recruiting CP patients aged between nine and 12 years.

## Conclusions

In conclusion, the available data indicates that the English version of the CP-QoL Child Self-Report questionnaire was effectively translated into Arabic and had excellent reliability and validity in evaluating the QoL of Saudi children with CP aged between nine and 12 years. The Arabic CP-QoL Child Self-Report questionnaire can be used to gain further understanding of the determinants of QoL, and it can be used to evaluate the effectiveness of different interventions for children with CP.
